# Дискордантные показатели инсулиноподобного фактора роста 1 (ИФР-1) и соматотропина (СТГ) в диагностике и мониторинге акромегалии

**DOI:** 10.14341/probl12791

**Published:** 2021-12-17

**Authors:** Е. Е. Сахнова, Е. Г. Пржиялковская, Ж. Е. Белая, Г. А. Мельниченко

**Affiliations:** Национальный медицинский исследовательский центр эндокринологии; Национальный медицинский исследовательский центр эндокринологии; Национальный медицинский исследовательский центр эндокринологии; Национальный медицинский исследовательский центр эндокринологии

**Keywords:** акромегалия, гормон роста, инсулиноподобный фактор роста-1, аденома гипофиза

## Abstract

Акромегалия — это редкое эндокринное заболевание, ассоциированное с множественными осложнениями и повышенной смертностью. Своевременная диагностика и адекватное лечение позволяют приблизить продолжительность жизни пациентов с акромегалией к общепопуляционному уровню. При скрининге, верификации диагноза и оценке эффективности различных методов лечения акромегалии используются следующие показатели: уровень гормона роста (соматотропного гормона, СТГ) в крови, как базальный, так и в ходе орального глюкозотолерантного теста (СТГ в ходе ОГТТ), и концентрация инсулиноподобного фактора роста-1 (ИФР-1). Вместе с тем в клинической практике до 39% пациентов с акромегалией имеют дискордантные результаты этих анализов. Ошибочная интерпретация не соответствующих друг другу лабораторных данных может приводить к гипердиагностике акромегалии или, наоборот, — несвоевременному выявлению заболевания, а также к избыточному назначению ненужных методов лечения или, в обратном случае, — длительному отсутствию адекватной терапии. В данном обзоре обсуждаются распространенность дискордантных результатов СТГ и ИФР-1 у пациентов с акромегалией; факторы, обуславливающие это расхождение, а также влияние несоответствия гормональных показателей на исходы лечения. Однозначного объяснения дискордантности уровней СТГ и ИФР-1 и руководства к ведению пациентов с акромегалией, имеющих такие результаты, к сожалению, в настоящее время не найдено. Специалисту крайне важно применять комплексный подход и учитывать все возможные факторы при интерпретации этих лабораторных показателей.

## Abstract (en)

Acromegaly is a rare endocrine disorder associated with multiple complications and increased mortality. Timely diagnosis and adequate treatment can bring the life expectancy of patients with acromegaly closer to the general population level. The tests for the diagnosis of acromegaly are measurement of both serum GH, and GH after oral glucose administration; serum insulin-like growth factor-1 (IGF-1). However, in clinical practice, up to 39% of patients with discordant results are found. The patients with discordant GH and IGF-1levels, are the most difficult to manage. This review discusses the prevalence of discordant GH and IGF-1 outcomes in patients with acromegaly; factors causing this discrepancy; the impact of hormone levels on treatment outcomes. Although endocrinologists are used to dealing with this discrepancy in clinical practice for many years, discordant patients’outcome remains uncertain and undefined The optimal treatment should be individually tailored for each patient, taking into account all clinical parameters.

## ВСТУПЛЕНИЕ

Акромегалия — это редкая, медленно прогрессирующая эндокринная патология, распространенность которой составляет 28–137 случаев на 1 млн населения, а заболеваемость колеблется от 2 до 11 случаев на 1 млн населения в год [1]. По данным Всероссийского регистра опухолей гипоталамо-гипофизарной области, пациенты с акромегалией встречаются с частотой 8,65 случая на 100 тыс. жителей Российской Федерации [2]. Акромегалия чаще всего обусловлена стойкой гиперсекрецией соматотропного гормона (СТГ) опухолью гипофиза, который, в свою очередь, стимулирует синтез инсулиноподобного фактора роста 1 (ИФР-1) в печени. Действие данных гормонов в органах и тканях приводит к развитию характерной клинической картины, различных системных осложнений и, как следствие, — повышенной смертности. За последние годы немалый прогресс достигнут в лечении акромегалии, которое включает нейрохирургическое вмешательство, медикаментозную терапию и лучевое воздействие. Своевременная диагностика и адекватное лечение позволяют приблизить продолжительность жизни пациентов с акромегалией к общепопуляционному уровню [3].

При скрининге, верификации диагноза и оценке эффективности различных методов лечения акромегалии используются следующие показатели: уровень СТГ в крови, как базальный, так и в ходе орального глюкозотолерантного теста (СТГ в ходе ОГТТ), и концентрация ИФР-1 [4]. Вместе с тем в клинической практике до 39% пациентов с акромегалией имеют дискордантные результаты этих анализов [5], что, безусловно, затрудняет выбор дальнейшей тактики ведения пациента. Ошибочная интерпретация не соответствующих друг другу лабораторных данных может приводить к гипердиагностике акромегалии или, наоборот, — несвоевременному выявлению заболевания, а также к избыточному назначению ненужных методов лечения или, в обратном случае, — длительному отсутствию адекватной терапии. Последствия таких неправильных решений — это снижение качества и продолжительности жизни пациентов и повышение экономических затрат системы здравоохранения.

В данном обзоре обсуждаются вопросы лабораторной диагностики и лабораторного мониторинга акромегалии на фоне различных методов лечения с акцентом на дискордантность результатов СТГ и ИФР-1. Описаны особенности физиологической и патологической секреции СТГ и ИФР-1, распространенность дискордантных результатов СТГ и ИФР-1 при акромегалии, факторы, обуславливающие это расхождение, а также влияние несоответствия гормональных показателей на исходы разных видов лечения.

Цель обзора — информировать специалистов о необходимости учитывать различные причины дискордантности лабораторных показателей в ходе принятия решения о ведении пациентов с акромегалией.

## ОСОБЕННОСТИ ФИЗИОЛОГИЧЕСКОЙ СЕКРЕЦИИ СТГ И ИФР-1

СТГ — это полипептидный гормон из семейства ростовых факторов, который синтезируется преимущественно в соматотрофах гипофиза, а также в других тканях, включая репродуктивную, лимфатическую системы и желудочно-кишечный тракт [6]. Для физиологической секреции СТГ характерна пульсация: выброс приблизительно каждые 3 ч и суточные колебания с преобладанием секреции в ночное время. Частота пульсации зависит от разных факторов, в первую очередь от пола и возраста. После рождения концентрация СТГ остается высокой очень недолго, затем снижается и поддерживается на одном уровне до пубертата, во время которого достигает пика и вырастает в 3 раза. С возрастом секреция СТГ падает: у мужчин — постепенно, приблизительно на 14% каждые 10 лет, у женщин — резко после достижения менопаузы [6].

Синтез и секреция СТГ регулируются в основном двумя гипоталамическими гормонами: рилизинг-гормоном соматолиберином, который повышает оба процесса, и соматостатином, который, взаимодействуя с соматостатиновыми рецепторами на соматотрофах, снижает выброс СТГ, но не влияет на его синтез. Кроме того, пептид грелин, который вырабатывается преимущественно в клетках желудка, стимулирует выброс СТГ [7]. ИФР-1 по принципу отрицательной обратной связи также подавляет секрецию гормона роста (рисунок).

**Figure:**
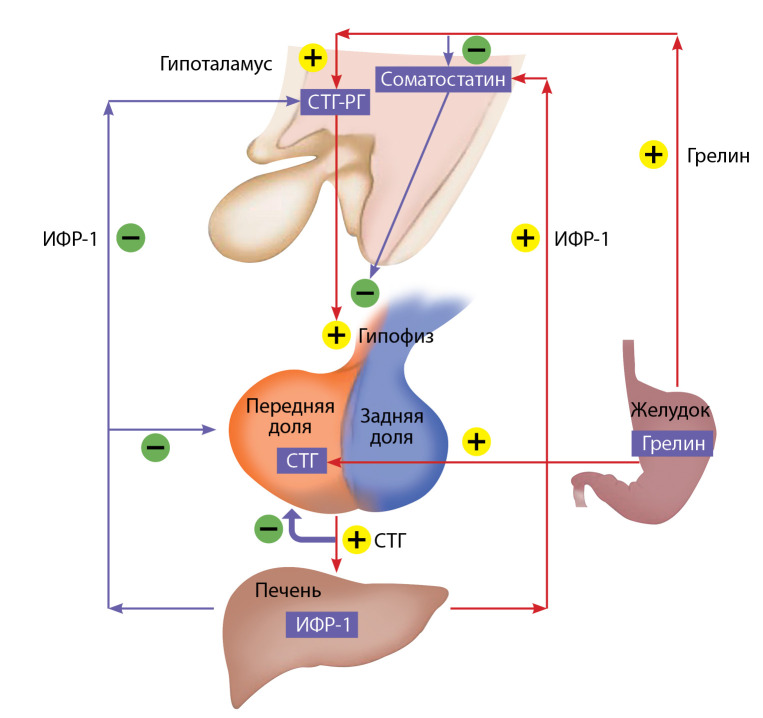
Рисунок. Схема физиологической регуляции синтеза и секреции соматотропного гормона и инсулиноподобного фактора роста 1. СТГ — соматотропный гормон, ИФР-1 — инсулиноподобный фактор роста 1, СТГ-РГ — соматолиберин.Стрелки указывают на стимулирующий (+) или угнетающий эффект (-) различных регуляторов.Адаптировано из: Wilkinson M., Imran S. Regulation of Growth Hormone Secretion. In Clinical Neuroendocrinology: An Introduction (pp. 134-153). Cambridge: Cambridge University Press; 2019. doi: 10.1017/9781108149938.009

ИФР-1 — это ростовой фактор, который синтезируется в основном в печени под действием СТГ. Концентрация ИФР-1 в крови определяется связывающими ИФР-1 белками. В отличие от СТГ уровень ИФР-1 не зависит от времени суток, приема пищи, физических упражнений или сна и вместе с этим отражает секрецию СТГ в течение нескольких предыдущих дней [8].

Уровень ИФР-1 в плазме увеличивается в 7 раз с момента рождения, достигая пика в период полового созревания [9]. Затем его концентрация быстро падает и к 20 годам составляет не более 50% максимального пубертатного уровня. Далее наблюдается постепенное снижение уровня ИФР-1 [10]. С одной стороны, это вызвано возрастными колебаниями уровня СТГ, с другой — может иметь генетическую обусловленность. Ученые обнаружили несколько полиморфизмов в гене ИФР-1 и ИФР-1-связывающих белков, объясняющих вариабельность концентраций ИФР-1 у здорового населения [11].

## ОБЩИЕ ВОПРОСЫ ЛАБОРАТОРНОЙ ДИАГНОСТИКИ И МОНИТОРИНГА АКРОМЕГАЛИИ

В связи с тем, что патологическая секреция СТГ и ИФР-1 лежит в основе развития акромегалии, именно эти показатели стали активно использовать для диагностики и оценки эффективности различных методов лечения этого заболевания. Поскольку секреция СТГ подвержена значительным колебаниям, случайное измерение СТГ имеет низкую диагностическую ценность и не рекомендуется для рутинного выявления акромегалии [8].

Золотым стандартом для скрининга и диагностики заболевания является измерение ИФР-1 в сыворотке крови [3][12].

В сомнительных случаях (при незначительном повышении уровня ИФР-1, отсутствии явной клинической картины акромегалии), в качестве второго метода диагностики используется определение СТГ в ходе ОГТТ. У здоровых людей повышение уровня глюкозы крови подавляет секрецию СТГ. Этот механизм изучен не полностью, предполагается опосредованное глюкозой увеличение секреции гипоталамического соматостатина [3]. Так, введение ингибитора ацетилхолинэстеразы (пиридостигмина), блокирующего выделение соматостатина в гипоталамусе, препятствует подавлению СТГ в ходе ОГТТ [13]. При бесконтрольной секреции гормона роста аденомой гипофиза чувствительность к физиологической регуляции утрачивается и, следовательно, подавления секреции СТГ не наблюдается [3].

Показано, что гипергликемия, обусловленная приемом 75 г безводной глюкозы, вызывает снижение уровня СТГ вплоть до минимально определяемых цифр у 94% здоровых лиц, но не у больных акромегалией [14]. В то же время до 41% лиц с акромегалией демонстрируют парадоксальный подъем уровня СТГ в ответ на гипергликемию. Данный феномен, по-видимому, объясняется повышенной экспрессией в СТГ-секретирующих аденомах гипофиза рецептора к глюкозозависимому инсулинотропному полипептиду [15].

Активную стадию акромегалии диагностируют, если хотя бы в одной точке, кроме базальной, отсутствует снижение уровня СТГ в ходе ОГТТ ниже 1 нг/мл, а при использовании высокочувствительных тестов — менее 0,4 нг/мл [16]. При наличии нарушений углеводного обмена возможно исследование ритма СТГ (5 точек в течение 2 ч с интервалом 30 мин) без приема глюкозы [12].

Безусловно, для окончательного подтверждения диагноза акромегалии, кроме данных лабораторного обследования, требуются наличие клинических проявлений и визуализация опухоли. К наиболее характерным симптомам акромегалии относятся изменения внешности (огрубение черт лица, увеличение кистей, стоп), отеки, головная боль, потливость, боли в суставах, онемение в кончиках пальцев, нарушения менструального цикла [3]. При топической диагностике методом выбора является магнитно-резонансная томография головного мозга. В случае противопоказаний возможно проведение компьютерной томографии. Отсутствие аденомы гипофиза при магнитно-резонансной томографии, выполненной с контрастированием на аппарате с высоким разрешением, требует дополнительных исследований для исключения эктопической формы акромегалии [12].

Оценка уровней СТГ и ИФР-1 применяется не только в диагностике, но и в мониторинге акромегалии. Поскольку высокие уровни СТГ и ИФР-1 ассоциированы с увеличением смертности пациентов с акромегалией, достижение нормализации СТГ (базального и в ходе ОГТТ) и ИФР-1 является основной целью различных методов лечения [17].

Снижение СТГ в ходе ОГТТ менее 1 нг/мл, а при использовании высокочувствительного метода определения — менее 0,4 нг/мл является критерием успешного нейрохирургического лечения в раннем послеоперационном периоде. При отсроченном наблюдении стойкая ремиссия акромегалии может быть установлена при нормализации уровня ИФР-1. На фоне медикаментозной терапии аналогами соматостатина рекомендуется исследование СТГ и ИФР-1, в то время как оценка СТГ в ходе ОГТТ мало информативна [17]. Пэгвисомант — антагонист рецептора к СТГ — в связи с особенностями своей структуры не способен снижать уровень СТГ, поэтому у пациентов, получающих лечение пэгвисомантом, концентрация ИФР-1 остается единственным критерием эффективности [18]. На фоне лучевой терапии, эффект которой может быть отсрочен во времени, для оценки наступления ремиссии рекомендовано периодическое исследование СТГ, СТГ в ходе ОГТТ и ИФР-1 [12]. Как и в случае с диагностикой, лабораторные показатели при мониторинге акромегалии должны оцениваться совместно с динамикой клинических проявлений и размеров опухоли. В настоящее время активно идет поиск предиктивных биомаркеров, при исследовании которых можно будет оценить вероятность развития ремиссии заболевания и предсказать чувствительность к разным видам лечения [19].

## ДИСКОРДАНТНЫЕ ПОКАЗАТЕЛИ ИФР-1 И СТГ

Итак, ИФР-1, СТГ и СТГ в ходе ОГТТ в настоящее время являются основными биомаркерами, которые используют для оценки активности заболевания у пациентов с акромегалией [16]. В большинстве случаев уровни СТГ и ИФР-1 совпадают, что указывает на ремиссию или активную стадию заболевания. Однако нередко уровни СТГ и СТГ в ходе ОГТТ расходятся с показателем ИФР-1, что значительно затрудняет интерпретацию результатов [20]. Дискордантные значения были продемонстрированы у 25% пациентов с акромегалией после проведенного нейрохирургического лечения [21]. В 35% случаев терапии аналогами соматостатина результаты СТГ не совпадали с данными ИФР-1 [22]. По данным E.O. Machado с коллегами, дискордантность СТГ и ИФР-1 может варьировать от 9,4 до 39% как на момент диагностики, так и при наблюдении пациентов с акромегалией [5].

Можно выделить два вида несоответствия лабораторных показателей: повышение уровня ИФР-1 при нормальных цифрах СТГ и повышение концентрации СТГ/отсутствие подавления СТГ в ходе ОГТТ при достижении референсных показателей ИФР-1.

Первый вариант дискордантности встречается в 5–62% случаев в зависимости от пороговых значений, установленных для СТГ [23]. Известно, что периферические ткани обладают повышенной чувствительностью к циркулирующему СТГ, поэтому даже «нормальная» или незначительно повышенная концентрация СТГ приводит к увеличению ИФР-1 [24].

Неоднократно показано, что многие пациенты (26–47% случаев) с клинической картиной акромегалии имеют «нормальный» уровень СТГ. A. Barkan предложил название «микромегалия» для данной формы заболевания. Пациентов с «микромегалией» отличают меньший размер опухоли гипофиза по сравнению с пациентами с повышенным уровнем СТГ, более низкий уровень ИФР-1 и пожилой возраст, что позволяет считать эту форму заболевания мягкой, но не начальной. У некоторых из таких пациентов может наблюдаться подавление СТГ в ходе ОГТТ ниже целевых значений [25]. Если диагностировать «микромегалию» не представляет трудностей в связи с яркой клинической картиной акромегалии и значительно повышенным уровнем ИФР-1 (более чем на 30% верхней границы нормы), то пациенты без явных симптомов заболевания с незначительным повышением уровня ИФР-1 и адекватным подавлением СТГ в ходе ОГТТ вызывают большие затруднения у специалистов, которые опасаются пропустить акромегалию в начальной стадии. Следует помнить, что у 5% населения может наблюдаться незначительно повышенный уровень ИФР-1. P.W. Rosario и M.R. Calsolari в течение 5 лет наблюдали пациентов с немного увеличенным уровнем ИФР-1 и достаточным подавлением СТГ в ходе ОГТТ, ни у одного из 42 пациентов не развилась акромегалия. Это позволило авторам утверждать, что снижение СТГ в ходе ОГТТ надежно исключает акромегалию [26].

Отсутствие подавления СТГ в ходе ОГТТ в сочетании с нормальным показателем ИФР-1 встречается у 9–39% пациентов. Хотя механизм такой дискордантности полностью не изучен, вероятно, уровень СТГ остается повышенным вследствие нарушенной регуляции секреции СТГ, которая не восстанавливается, несмотря на успешное лечение акромегалии [23].

Затруднения, которые возникают при интерпретации противоречивых данных лабораторных исследований, приводят к тому, что многие специалисты выбирают исследование ИФР-1 как единственный маркер активности акромегалии. Может, не следует вовсе оценивать уровень СТГ при акромегалии? E.H. Oldfield с коллегами показали, что при акромегалии у пациентов с одинаковым уровнем СТГ могут быть совершенно разные показатели ИФР-1 как до, так и после лечения, и нет линейной зависимости между этими показателями. И хотя ИФР-1 является интегрированным показателем повышенной секреции и действия СТГ, аденома гипофиза секретирует именно СТГ, а не ИФР-1. Авторы считают, что для того, чтобы оценить активность опухоли, необходимо обязательно исследовать уровень СТГ у пациентов с акромегалией [27].

## ЛАБОРАТОРНЫЕ ПРИЧИНЫ ДИСКОРДАНТНОСТИ СТГ И ИФР-1

При отсутствии очевидного объяснения противоречивых результатов СТГ и ИФР-1 необходимо помнить о наличии двух основных причин такой дискордантности: влияния лабораторных и биологических факторов.

Известно, что в сыворотке крови СТГ циркулирует в виде множества различных изоформ, фрагментов и молекулярных комплексов (гомо- и гетеродимеров и олигомеров). Изоформа весом 22 kDa составляет более 90% всех изоформ и лучше всего отражает общую секрецию СТГ гипофизом. Однако в реальной клинической практике многие лаборатории используют тестовые системы, распознающие более широкий спектр изоформ или вовсе не имеющие изоформной специфичности, что, в свою очередь, часто приводит к неверной интерпретации лабораторных показателей [28].

За последние десятилетия лабораторное тестирование СТГ прошло путь от относительно неспецифического радиоиммунного анализа до современного хемилюминесцентного высокочувствительного метода определения [8]. Эволюция аналитических методов привела к ужесточению целевых значений СТГ, что, естественно, отразилось на частоте дискордантных результатов. Так, процент несоответствия лабораторных показателей у пациентов, которым диагноз акромегалии был поставлен за последние 10 лет, оказался значительно выше, чем в случаях установления диагноза ранее 2011 г. В первую очередь это обусловлено недоступностью высокочувствительных тестов для определения концентрации гормонов и менее строгими критериями диагностики [25]. Так, до 1990-х гг. ремиссии акромегалии после проведенного лечения соответствовал базальный СТГ ниже 5 нг/мл [29]. С появлением новых методов анализа были предложены более низкие пороговые значения. В середине-конце 1990-х гг. базальный СТГ ниже 2,5 нг/мл и СТГ в ходе ОГТТ менее 2 нг/мл стали критериями успешного лечения [30]. В начале 2000-х гг. случайный уровень СТГ ниже 0,4 нг/мл или СТГ в ходе ОГТТ менее 1 нг/мл вместе с нормальным показателем ИФР-1 в соответствии с полом и возрастом были определены как критерии исключения акромегалии [31]. Десять лет спустя данные показатели были пересмотрены, и целевыми показателями после проведенного лечения стали считать случайный базальный уровень СТГ ниже 1 нг/мл и подавление СТГ в ходе ОГТТ менее 0,4 нг/мл при использовании высокочувствительных тестов в сочетании с нормальным ИФР-1. До настоящего времени, в том числе согласно международному консенсусу по диагностике и лечению акромегалии 2019 г., данные критерии не изменились [16].

Проблемы тест-систем в той же степени касаются и оценки уровня ИФР-1. Согласно данным A. Pokrajac et al. (2007), концентрация ИФР-1 у одного и того же больного колебалась в разных лабораториях в широком диапазоне, от 24,3 до 60,9 нмоль/л [32]. В крупном французском исследовании, проведенном среди 911 здоровых добровольцев, уровень ИФР-1, измеренный в 6 различных лабораториях, не совпадал в 38–70% случаев [33].

Тест-системы для определения уровня ИФР-1 также со временем были усовершенствованы. Переход от использования поликлональных антител к иммуноанализу на основе моноклональной сыворотки повысил не только чувствительность, но и специфичность тестов при исследовании ИФР-1. Из-за наличия белков, связывающих ИФР-1, применение высокочувствительных тестов играет ключевую роль в определении истинной концентрации данного показателя [34].

Современные подходы к определению ИФР-1 включают электрохемилюминесцентный или хемилюминесцентный иммуноанализ, при которых используют антитела для связывания антигенов, а также жидкостную хромато-масс-спектрометрию, определяющую концентрацию гормона на основе молекулярной массы за счет ионизации компонентов [35].

При сравнении данных методов в 24% было выявлено несоответствие лабораторных показателей. При масс-спектрометрии измеряется 1, наиболее распространенный ион ИФР-1, исключая варианты с другой молекулярной массой. В свою очередь, при иммуноанализе все типы ИФР-1 одинаково связываются антителами. Поэтому использование иммуноанализа приводило к получению более высоких уровней ИФР-1, а при масс-спектрометрии отмечались ложнозаниженные результаты [34].

Кроме того, в разных лабораториях варьируют референсные интервалы, определение которых напрямую зависит от пола, индекса массы тела, региона проживания, приема фармакологических препаратов и наличия сопутствующей патологии у исследуемой популяции. Усредненный диапазон показателей ИФР-1, характеризующий «норму», должен устанавливаться на основе анализа результатов большой когорты пациентов, отобранных по определенным критериям [8].

Таким образом, рекомендуется использовать одну и ту же надежную лабораторию для оценки ИФР-1 на протяжении всего периода наблюдения за пациентом с акромегалией [35].

## БИОЛОГИЧЕСКИЕ ПРИЧИНЫ ДИСКОРДАНТНОСТИ СТГ И ИФР-1

При интерпретации лабораторных показателей необходимо учитывать, что многие физиологические и патологические состояния, гормоны и лекарственные препараты влияют на уровни СТГ и ИФР-1 в сыворотке крови (табл. 1).

**Table:** Таблица 1. Влияние биологических факторов на концентрации СТГ и ИФР-1

Биологический фактор	СТГ	ИФР-1
Ожирение	↓	↓
Голодание	↑	↓
Нервная анорексия	↑	↓
Стресс	↑	↑↓
Пубертат	↑	↑
Беременность	↑	↓
Сон	↑	↑↓
Спорт	↑	↑↓
Сахарный диабет 1 типа	↑	↓
Почечная недостаточность	↑	↓
Печеночная недостаточность	↑	↓
Гипотиреоз	↓	↓
Гипертиреоз	↑	↑
Тестостерон	↑	↑
Оральные эстрогены	↑	↓
Системное воспаление	↑	↓
Беременность	↑	↑↓

В частности, такие состояния, как пубертат, нервная анорексия, прием эстрогенов, гипертиреоз, сахарный диабет, ожирение, почечная недостаточность и хронический гепатит ассоциированы с отсутствием подавления СТГ в ходе ОГТТ [36].

Так, во время пубертата значительно повышаются уровни СТГ и ИФР-1, что затрудняет диагностику акромегалии у подростков. Такие состояния, как боль, занятия спортом, стресс, нервная анорексия, стимулируют выброс СТГ [8].

Значительное влияние на систему СТГ/ИФР-1 оказывают половые стероиды. Было показано, что тестостерон усиливает спонтанную секрецию СТГ. Напротив, эстрогены ослабляют действие СТГ, тем самым снижая продукцию ИФР-1. Рекомендовано с осторожностью интерпретировать результаты исследования СТГ и ИФР-1 у женщин, получающих оральные эстрогены в качестве заместительной гормональной терапии [7].

Беременность приводит к увеличению секреции СТГ и ИФР-1 из-за выработки большего количества биологически активного плацентарного гормона роста [28], однако концентрация ИФР-1 может снижаться при акромегалии во время беременности за счет высокого содержания эстрогенов.

Недостаток тиреоидных гормонов снижает концентрации СТГ и ИФР-1 в сыворотке крови [37]. При гипертиреозе, наоборот, усиливается выброс СТГ [38]. Избыток тиреоидных гормонов также повышает содержание ИФР-1, однако его биологическая активность снижается в связи с увеличением концентрации ИФР-1-связывающих белков [37].

У пациентов с сахарным диабетом 1 типа наблюдается повышение секреции гормона роста. При этом дефицит инсулина в воротной вене препятствует влиянию СТГ на печень и ведет к снижению продукции ИФР-1 [39]. Данные о влиянии сахарного диабета 2 типа на секрецию СТГ и ИФР-1 противоречивы. Спонтанная секреция СТГ может быть повышена, не изменяться или снижаться. Эти различия в большинстве случаев обусловлены наличием или отсутствием сопутствующего ожирения. Исследования с участием пациентов, имеющих индекс массы тела более 30 кг/м2, продемонстрировали снижение как спонтанной, так и стимулированной секреции СТГ у данной группы пациентов [40].

При снижении почечной функции может повышаться уровень СТГ вследствие снижения почечной деградации СТГ и устойчивости рецепторов к его воздействию. В дополнение к этому снижается клиренс ИФР-1-связывающих белков, что приводит к снижению биологической активности ИФР-1 [41].

При печеночной недостаточности наблюдается снижение синтеза ИФР-1 в печени, что влечет за собой увеличение секреции СТГ за счет механизма отрицательной обратной связи [42].

Ложное снижение уровня ИФР-1 (вплоть до нормальных значений, соответствующих полу и возрасту) возникает при голодании и злоупотреблении алкоголем [16].

## ДИСКОРДАНТНОСТЬ СТГ И ИФР-1 ПОСЛЕ НЕЙРОХИРУРГИЧЕСКОГО ЛЕЧЕНИЯ АКРОМЕГАЛИИ

Большинству пациентов с акромегалией трансназальная транссфеноидальная аденомэктомия рекомендована в качестве первого метода лечения [3]. Уровень ИФР-1 для надежного определения ремиссии заболевания должен оцениваться как минимум через 3 мес после операции, так как для нормализации данного показателя необходимо время [43]. СТГ в ходе ОГТТ в послеоперационном периоде менее 1 нг/мл, а при использовании высокочувствительного метода исследования — менее 0,4 нг/мл является критерием послеоперационной ремиссии [16].

Однако если пациенты до операции получали аналоги соматостатина, эти данные следует интерпретировать с осторожностью [44].

Тщательный мониторинг требуется пациентам, имеющим дискордантные показатели СТГ и ИФР-1 через 3 мес после операции. Чаще всего наблюдается подавление СТГ при сохранении повышения уровня ИФР-1, но и обратное расхождение тоже встречается. Последний консенсус по лечению акромегалии рекомендует ориентироваться в большей степени на уровень ИФР-1 у таких пациентов [16]. Предполагается, что полиморфизм рецептора гормона роста (d3-GHR) влияет на дискордантность СТГ и ИФР-1 во время лечения акромегалии. Так, в исследовании A. Bianchi и соавт. 20% пациентов имели несоответствие лабораторных показателей в послеоперационном периоде, и у 71% из них был обнаружен d3-GHR [45].

В исследование, опубликованное в 2004 г., были включены 110 пациентов с акромегалией после проведенной транссфеноидальной аденомэктомии. Из них 76 человек имели полную ремиссию заболевания. Однако у 34% из них наблюдались дискордантные показатели (ИФР-1 в пределах референса при отсутствии адекватного подавления СТГ в ходе ОГТТ). Авторы продемонстрировали, что при длительном наблюдении за этой группой пациентов нецелевой уровень СТГ был связан с более высокой частотой рецидива акромегалии [46].

В ретроспективное исследование T. Graillona и соавт., опубликованное в 2020 г., были включены 167 пациентов, оперированных по поводу впервые выявленной акромегалии в период с 1997 по 2014 гг. Данная группа не получала терапию аналогами соматостатина за месяц до нейрохирургического лечения. Оценка показателей ИФР-1, СТГ в ходе ОГТТ проводилась в раннем послеоперационном периоде (7 дней после операции), через 3 мес, через год и затем ежегодно.

Пациенты были разделены на 4 группы в зависимости от соответствия уровней ИФР-1 и СТГ/СТГ в ходе ОГТТ и необходимости в дополнительном лечении. Очевидно, что наиболее стойкая ремиссия была отмечена у пациентов с нормальным уровнем ИФР-1 и адекватным подавлением СТГ в ходе ОГТТ (менее 0,4 нг/мл) или средним уровнем СТГ менее 1 нг/мл. А самой трудной для наблюдения стала группа пациентов с дискордантными показателями СТГ и ИФР-1, которая наблюдалась без дополнительного лечения акромегалии. Тем не менее 50% пациентов с несовпадающими данными СТГ и ИФР-1 достигли стойкой послеоперационной ремиссии заболевания в течение года без дополнительной терапии, у 10% была однозначно установлена активная стадия, у оставшихся пациентов дискордантность сохранялась более 1 года после нейрохирургического лечения. Вопрос о тактике ведения таких пациентов остается на данный момент нерешенным [47].

## ДИСКОРДАНТНОСТЬ СТГ И ИФР-1 НА ФОНЕ МЕДИКАМЕНТОЗНОЙ ТЕРАПИИ АКРОМЕГАЛИИ

Медикаментозное лечение в качестве 2-й линии терапии рекомендовано всем пациентам, не достигшим ремиссии акромегалии после оперативного вмешательства. Как основной метод лечения консервативная терапия может быть предложена пациентам, имеющим противопоказания к транссфеноидальной аденомэктомии, или в случае отказа от хирургического лечения [3].

В исследовании J. Dal, опубликованном в 2017 г., показано, что повышенный уровень базального СТГ и отсутствие его подавления в ходе ОГТТ преобладают у пациентов, получающих терапию аналогами соматостатина, по сравнению с послеоперационными пациентами [48]. К сходным выводам пришли и J.D. Carmichael с коллегами в своей работе, включившей 166 пациентов с акромегалией. Противоречивые результаты ИФР-1 и СТГ в ходе ОГТТ наблюдались у 33% больных после проведенного нейрохирургического лечения, не получающих медикаментозную терапию, у 48% пациентов, находящихся на терапии аналогами соматостатина, и у 18% на фоне лечения агонистами дофаминовых рецепторов. На фоне терапии аналогами соматостатина преобладали несоответствие нормального уровня ИФР-1 и отсутствие подавления СТГ в ходе ОГТТ (42%) по сравнению с повышенным ИФР-1 и адекватным снижением СТГ (6%) [44].

В то время как другие авторы на основе данных бельгийского регистра пациентов с акромегалией, наоборот, чаще демонстрировали высокие цифры ИФР-1 при нормальных показателях СТГ (24%) по сравнению с обратной дискордантностью (11%) [22]. Различные пороговые значения СТГ для установления активности заболевания и неоднородные выборки пациентов могут объяснить широкий разброс частоты и преобладание одного или другого типа дискордантности в разных работах. Тем не менее целесообразность применения СТГ в ходе ОГТТ на фоне терапии аналогами соматостатина подвергается сомнению в последнее время [44].

Дискордантные результаты обычно приводят к увеличению дозы аналогов соматостатина и, соответственно, к улучшению биохимического контроля акромегалии [48]. Однако нельзя исключить, что при нормализации уровня ИФР-1 на фоне терапии аналогами соматостатина сохраняется избыточная секреция СТГ, что требует другого терапевтического подхода [49].

Вторая линия медикаментозной терапии акромегалии — это применение антагониста рецептора СТГ (пэгвисоманта). Препарат показан в первую очередь пациентам, у которых хирургическое лечение оказалось неэффективным и продемонстрирована резистентность к терапии аналогами соматостатина [3]. Пэгвисомант, блокируя действие эндогенного СТГ на рецепторном уровне, снижает продукцию ИФР-1, тем самым уменьшая выраженность клинических проявлений и развитие осложнений, связанных с акромегалией. Так как препарат напрямую не влияет на повышенную секрецию гормона роста, измерение уровня СТГ, базального и в ходе ОГТТ, и исследование причин выявления дискордантных результатов в данном случае нецелесообразно [50]. Кроме того, у пациентов, получающих пэгвисомант, невозможно измерить истинную концентрацию СТГ. В зависимости от тест-системы наличие пэгвисоманта в сыворотке крови может приводить к ложноположительным или ложноотрицательным показаниям СТГ [8].

## ЗАКЛЮЧЕНИЕ

В клинической практике интерпретация противоречивых лабораторных данных СТГ и ИФР-1 часто осложняет специалистам выбор дальнейшей тактики ведения пациентов с акромегалией. К сожалению, на данный момент нет единого объяснения дискордантности СТГ и ИФР-1 и единого подхода к принятию врачебного решения. Понимание того, что пороговые значения и референсные интервалы гормональных показателей напрямую зависят от аналитических методов, используемых различными лабораториями, должно способствовать созданию единых критериев для оценки уровня исследуемых показателей. Кроме того, специалисту необходимо учитывать широкий спектр физиологических и патологических факторов, лекарственных препаратов, влияющих на уровни СТГ и ИФР-1.

Таким образом, при ведении пациентов с акромегалией особенно важно применение комплексного подхода: важно не только верно интерпретировать лабораторные показатели, но и принимать во внимание такие параметры, как возраст пациента, размер опухоли, степень ее инвазии, наличие осложнений и сопутствующей патологии. В связи с этим актуальным остается вопрос поиска новых биомаркеров, отражающих биохимическую активность заболевания и способных предсказать чувствительность к различным видам лечения.

## ДОПОЛНИТЕЛЬНАЯ ИНФОРМАЦИЯ

Источники финансирования. Поисково-аналитическая работа и подготовка статьи проведены за счет гранта Российского научного фонда (проект №19-15-00398).

Конфликт интересов. Авторы декларируют отсутствие явных и потенциальных конфликтов интересов, связанных с публикацией настоящей статьи.

Участие авторов. Все авторы внесли значимый вклад в написание статьи, прочли и одобрили финальный вариант рукописи до публикации.
